# Long-term Psychological Impact of the COVID-19 Pandemic: A Cross-Sectional Survey among Students and Technical Staff in Health Care Institutions

**DOI:** 10.12688/f1000research.169634.1

**Published:** 2025-09-03

**Authors:** Vignesh Kamath, Ann Sales, Umesh Pai, Srikant N., Abhinav Bhargava, Vaishnavi Jalaj

**Affiliations:** 1 Department of Prosthodontics and Crown & Bridge, Manipal College of Dental Sciences Mangalore, Manipal Academy of Higher Education, Manipal, Karnataka, 576104, India; 2 Department of Oral Pathology and Microbiology, Manipal College of Dental Sciences Mangalore, Manipal Academy of Higher Education, Manipal, Karnataka, 576104, India; 3 Department of Public Health Dentistry, ITS Centre for Dental Studies and Research, Ghaziabad, Uttar Pradesh, 201007, India; 4 Manipal College of Dental Sciences Mangalore, Manipal Academy of Higher Education, Manipal, Karnataka, 576104, India

**Keywords:** Anxiety, Coronavirus, Depression, DASS scale, Technical staff, Long-term impact

## Abstract

**Background:**

The aim of this study is to evaluate the long-term psychological impact of the COVID-19 pandemic on students and technical personnel at healthcare institutions, with a focus on levels of depression, anxiety, and stress.

**Methods:**

A total of 250 participants were included, comprising 130 students from health sciences and 120 individuals from technical fields. In total, 164 participants were females, and 192 participants were aged ≤20 years. A cross-sectional study was conducted using a self-administered electronic questionnaire. The Depression, Anxiety, and Stress Scale (DASS-21), followed by the Impact of Event Scale-Revised (IES-R), was used to retrospectively assess psychological distress linked to the pandemic. The data were analysed via the chi-square test.

**Results:**

DASS severity ratings revealed that 41.3% of participants aged ≤20 years experienced severe or extremely severe anxiety, which was statistically significant (p = 0.013). The stress scores were also significantly higher in this age group (p = 0.004). Binary logistic regression analysis indicated that younger age and female sex were significantly associated with increased odds of depression and stress. Males had comparatively lower odds of experiencing psychological distress. Participants from technical fields demonstrated higher odds of anxiety and stress, although not of depression.

**Conclusion:**

The long-term psychological impact of the COVID-19 pandemic appears to be more pronounced among younger individuals, females, and technical staff in healthcare institutions. Although students may have greater awareness about the disease, this awareness may contribute to heightened psychological distress.

## Introduction

The COVID-19 pandemic, which was declared a global health emergency by the World Health Organization, has had a profound and lasting impact on global health and quality of life. The virus’s high infectivity, rapid transmission, and initially unclear mechanism of action led to widespread fear, uncertainty, and psychological distress. In response, countries around the world implemented stringent public health measures, including lockdowns, international travel restrictions, physical distancing mandates, and compulsory mask use, all of which significantly altered daily routines and social interactions. ^1^

These drastic changes in social norms have contributed to considerable psychological stress, particularly among individuals in healthcare settings. Even in high-awareness environments such as medical institutions, students and trainees face heightened mental strain fueled by misinformation, fear of transmitting the virus to family members, academic disruption, and isolation. These factors lead to difficulty concentrating, disrupted sleep patterns, and reduced opportunities for social interaction, further exacerbating feelings of anxiety and stress. ^2, 3^ For many students—particularly those unable to relocate or lacking nearby support systems—quarantine and physical distancing intensified symptoms of anxiety, depression, and trauma-related behaviors, including obsessive tendencies and posttraumatic stress. ^4, 5^ Similar psychological effects have been observed among technical staff, who often lack adequate resources or understanding to manage the crisis, thereby increasing their vulnerability to mental health challenges. ^6^

In response to these widespread psychological consequences, the importance of implementing mental health support systems—such as counselling and resilience-building programs—has become increasingly apparent. ^7, 8^ Educational institutions also recognized the urgent need for policy adaptations to accommodate the shift from in-person to virtual learning, as well as to support the psychological well-being of students and staff. ^9^

To develop effective mental health strategies, it is essential to understand the specific stressors and mental health outcomes that continue to affect these populations during the postpandemic period. This study was designed to retrospectively assess the long-term psychological impact of the COVID-19 pandemic on students and technical staff in healthcare institutions. By identifying persistent symptoms of depression, anxiety, and stress, this study aims to contribute valuable insights for future mental health interventions and policy planning.

## Materials & Methods

This cross-sectional study was conducted among a total of 250 participants, comprising 130 students from health sciences and 120 technical staff (nontaching personnel) working in healthcare institutions. Among the total participants, 164 were female, and 192 were aged ≤20 years. Ethical approval for the study was obtained from the Institutional Ethics Committee (IEC:231/2021).

To retrospectively assess the psychological effects of the COVID-19 pandemic, a self-administered electronic questionnaire was used. The questionnaire was created via Google Forms, enabling contactless data collection.

This study employed two widely used and previously validated instruments: the Depression, Anxiety and Stress Scale – 21 Items (DASS-21) ^10^ and the Impact of Event Scale – Revised (IES-R). ^11^ The DASS-21 is a shortened version of the original DASS-42, developed and validated by Lovibond & Lovibond (1995) to assess symptoms of depression, anxiety, and stress [Link to DASS manual]. The IES-R, developed and validated by Weiss & Marmar (1997), is a well-established measure for assessing subjective distress caused by traumatic events [Link to IES-R scale]. As both scales are universally recognized and psychometrically validated, no further validation was undertaken in the present study.

Participant lists were obtained from student records and the human resources department. A systematic sampling method was applied, selecting every third individual from the compiled lists. The survey link was distributed individually to participants via social media messaging platforms. A consent section was included at the beginning of the form, where participants responded to a yes/no question to indicate their willingness to participate in the study.

The first section of the questionnaire collected sociodemographic information, including age, sex, and occupational category (student or technical staff ). Additional items were used to assess participants’ knowledge of COVID-19, personal diagnosis status, and contact history with individuals who tested positive or were suspected of having the virus.

The second section focused on assessing the long-term psychological impact of the pandemic. It comprises two standardized tools: the 21-item Depression, Anxiety, and Stress Scale (DASS-21), followed by the 22-item Impact of Event Scale–Revised (IES-R). Responses on the DASS-21 were scored on a 4-point Likert scale ranging from 0 (did not apply to me at all) to 3 (applied to me very much or most of the time). Total scores for depression, anxiety, and stress were calculated and categorized as normal, mild, moderate, severe, or extremely severe, following the established scoring guidelines. The methodology was adapted from a similar study conducted by Hakami Z et al. ^7^

The IES-R was used to assess trauma-related distress and included questions related to the psychological impact of stressful life events. The scores were interpreted in three categories: mild (24–32), moderate (33–36), and severe (≥37) psychological impact. Associations between mental health outcomes and demographic variables (age ≤20 vs. >20 years, sex, and field of work or study) were analysed via the chi-square test.

## Results

A greater proportion of participants in the ≤20 years age group, females, and those from the health sciences field exhibited severe or extremely severe depression on the basis of DASS-21 severity ratings; however, these differences were not statistically significant. A similar pattern was observed for anxiety. Notably, 41.3% of individuals aged ≤20 years reported severe or extremely severe anxiety, which was statistically significant (p = 0.013). The stress levels were also significantly greater in this age group (p = 0.004). While females and participants from the technical field presented elevated stress scores, these findings were not statistically significant.

IES-R scoring revealed that most participants across all subgroups had scores within the normal range. Nonetheless, slightly higher proportions of individuals in the ≤20 years group, females, and technical staff fell into the category of “high enough to be immunosuppressive.” These differences, however, did not reach statistical significance [ Table 1].

**Table 1.** Chi square test to compare the DASS severity rating and IES-R scoring among age groups, gender, and field of teaching.

	Categories	N	AGE		Gender		Field	
			<=20 years (N (%))	>20 years (N (%))	Female (N (%))	Male (N (%))	Health Science (N (%))	Technical (N (%))
Dass Severity Rating-Depression	Normal	118	21 (36.2)	97 (50.5)	76 (46.3)	42 (48.8)	59 (45.4)	59 (49.2)
	Mild	30	7 (12.1)	23 (12)	20 (12.2)	10 (11.6)	17 (13.1)	13 (10.8)
	Moderate	62	17 (29.3)	45 (23.4)	40 (24.4)	22 (25.6)	33 (25.4)	29 (24.2)
	Severe	22	7 (12.1)	15 (7.8)	16 (9.8)	6 (7)	13 (10)	9 (7.5)
	Extremely Severe	18	6 (10.3)	12 (6.2)	12 (7.3)	6 (7)	8 (6.2)	10 (8.3)
	**Chi square (P value)**		**4.508 (0.342)**		**0.626 (0.96)**		**1.343 (0.854)**	
Dass Severity Rating-Anxiety	Normal	114	20 (34.5)	94 (49)	68 (41.5)	46 (53.5)	62 (47.7)	52 (43.3)
	Mild	14	2 (3.4)	12 (6.2)	9 (5.5)	5 (5.8)	7 (5.4)	7 (5.8)
	Moderate	53	12 (20.7)	41 (21.4)	35 (21.3)	18 (20.9)	28 (21.5)	25 (20.8)
	Severe	28	6 (10.3)	22 (11.5)	19 (11.6)	9 (10.5)	14 (10.8)	14 (11.7)
	Extremely Severe	41	18 (31)	23 (12)	33 (20.1)	8 (9.3)	19 (14.6)	22 (18.3)
	**Chi square (P value)**		**12.592 (0.013)**		**5.894 (0.207)**		**0.868 (0.929)**	
Dass Severity Rating-Stress	Normal	154	28 (48.3)	126 (65.6)	98 (59.8)	56 (65.1)	83 (63.8)	71 (59.2)
	Mild	35	6 (10.3)	29 (15.1)	23 (14)	12 (14)	17 (13.1)	18 (15)
	Moderate	28	9 (15.5)	19 (9.9)	17 (10.4)	11 (12.8)	15 (11.5)	13 (10.8)
	Severe	28	14 (24.1)	14 (7.3)	22 (13.4)	6 (7)	13 (10)	15 (12.5)
	Extremely Severe	5	1 (1.7)	4 (2.1)	4 (2.4)	1 (1.2)	2 (1.5)	3 (2.5)
	**Chi square (P value)**		**15.47 (0.004)**		**3.107 (0.54)**		**1.051 (0.902)**	
IES-R Scoring Consequence	Normal	129	26 (44.8)	103 (53.6)	85 (51.8)	44 (51.2)	75 (57.7)	54 (45)
	Clinical Concern	22	3 (5.2)	19 (9.9)	12 (7.3)	10 (11.6)	11 (8.5)	11 (9.2)
	Probable Diagnosis of PTSD	13	5 (8.6)	8 (4.2)	9 (5.5)	4 (4.7)	5 (3.8)	8 (6.7)
	High Enough to be Immunosuppresive	86	24 (41.4)	62 (32.3)	58 (35.4)	28 (32.6)	39 (30)	47 (39.2)
	**Chi square (P value)**		**4.569 (0.206)**		**1.401 (0.705)**		**4.462 (0.216)**	

Binary logistic regression analysis demonstrated that age and sex were significantly associated with psychological outcomes. Participants aged ≤20 years had significantly greater odds of experiencing depression (OR: 1.86; p = 0.048) and stress (OR: 2.05; p = 0.022). Compared with females, males had significantly lower odds of depression, anxiety, and stress (OR: 0.55; p = 0.034).

Interestingly, individuals from technical fields showed increased odds of anxiety and stress, but not depression. Greater awareness of COVID-19, knowing someone infected, or having contact with a suspected case were associated with higher odds of psychological distress, but these associations were not statistically significant [ Table 2].

**Table 2.** Binary logistic regression analysis to assess the odds of depression, anxiety or stress adjusted for age, sex, field, awareness and association with COVID 19 infected individuals.

	Prediction of depression			Prediction of anxiety			Prediction of stress		
	Beta	OR (95% CI)	P value	Beta	OR (95% CI)	P value	Beta	OR (95% CI)	P value
AGE (<=20 years compared to >20 years)	0.622	1.86 (1.01, 3.45)	**0.048**	0.624	1.87 (0.99, 3.51)	0.053	0.72	2.05 (1.11, 3.81)	**0.022**
Gender (Male Compared to female)	-0.088	0.92 (0.54, 1.57)	0.748	-0.595	0.55 (0.32, 0.96)	**0.034**	-0.358	0.7 (0.39, 1.24)	0.221
Field (Technical compared to Health Sciences)	-0.169	0.85 (0.51, 1.41)	0.521	0.277	1.32 (0.78, 2.24)	0.303	0.246	1.28 (0.74, 2.2)	0.375
Awareness (Ref No awareness)	0.541	1.72 (0.27, 10.82)	0.564	0.618	1.86 (0.29, 12.06)	0.517	-0.17	0.84 (0.13, 5.55)	0.86
Known someone who infected with Covid19 (Ref No)	0.165	1.18 (0.69, 2.03)	0.552	0.396	1.49 (0.85, 2.59)	0.163	0.512	1.67 (0.95, 2.93)	0.074
Contacted with someone who suspected with Covid19 (Ref No)	0.216	1.24 (0.51, 3.03)	0.635	0.677	1.97 (0.75, 5.17)	0.169	0.749	2.12 (0.86, 5.23)	0.105
Have first degree relatives whom are medical field related professional (Ref No)	-0.017	0.98 (0.59, 1.63)	0.949	0.153	1.17 (0.7, 1.96)	0.562	0.44	1.55 (0.91, 2.65)	0.106
Constant	-0.52		0.586	-0.771		0.428	-0.965		0.326

## Discussion

The COVID-19 pandemic represents one of the most severe public health crises in modern history. In addition to its devastating mortality toll, the pandemic has had a profound psychological impact on populations worldwide. Individuals across all age groups and professions experienced heightened levels of despair, anxiety, and stress due to prolonged uncertainty, isolation, and social disruption. ^12, 13^ Measures such as social distancing, remote work, and online education radically altered daily life and revealed underlying inequalities, disproportionately affecting marginalized and vulnerable communities. ^9^ These challenges have continued to influence mental well-being, even in the postpandemic period, underscoring the ongoing need to prioritize mental health support at the institutional and governmental levels.

Depression, anxiety, and stress, while often interrelated, manifest differently across individuals and contexts. ^2, 14^ In this study, each variable was evaluated separately via standardized tools, taking into account age, sex, and professional background. The analysis revealed that participants aged ≤20 years, females, and individuals from the health sciences presented higher proportions of severe and extremely severe depression, although these findings were not statistically significant. These results are consistent with previous research by Ansari et al., who reported elevated psychological symptoms in young adult populations during the pandemic. Similarly, Chi et al. reported a high prevalence of posttraumatic, depressive, and anxiety symptoms among Chinese university students. ^15^

A key observation in the present study was the significantly higher rate (41.3%) of severe or extremely severe anxiety among participants aged ≤20 years (p = 0.013). This trend was more pronounced among females and those in the health sciences. These findings are in line with those of studies by Verma et al. and Sundarasen et al., which documented widespread psychological distress in similar demographic groups during and after the pandemic. ^9, 16^ Notably, dental students in their clinical years—facing academic disruption and uncertainty—were reported to have higher anxiety levels than preclinical students did, a pattern echoing the results of the present study. ^4^

Our stress scale and IES-R results further support these trends, which aligns with findings from Changwon Son et al., who highlighted elevated mental health symptoms among university students during the pandemic. ^4^ A nationwide survey by Hakami et al. in Saudi Arabia reported significantly greater depression, anxiety, and stress among dental students, particularly females and those living alone. ^7^

Binary logistic regression in our study confirmed that age and sex were significantly associated with increased odds of depression and stress. These results mirror those of Debowska et al., who reported greater psychological distress among female students and those aged 18–24 years than among older students. ^16– 18^ Khan et al. also reported increased anxiety and fear among healthcare workers, particularly females, during postings in COVID-19 wards. ^19^

While the study provides valuable insights, it is not without limitations. The use of an e-questionnaire allows for safe and efficient data collection, but it may not fully capture the depth of participants’ emotional states. Furthermore, the tools used—while validated—were not specifically designed to assess COVID-19-related psychological distress, such as the Coronavirus Anxiety Scale (CAS), which may have provided more targeted insights. ^10, 20^ Additionally, the study’s sample primarily comprised individuals from medical backgrounds, which may limit the generalizability of the findings.

Another limitation is the lack of assessment of potential confounding factors such as socioeconomic status, family dynamics, substance use, and preexisting mental health conditions. These variables can significantly influence psychological outcomes. A more comprehensive multivariate analysis incorporating these factors would offer a deeper understanding of the long-term mental health implications for healthcare students and professionals.

Despite these constraints, the findings of this study contribute to the growing body of evidence highlighting the sustained psychological burden of the COVID-19 pandemic. As we move forward, it is essential to implement ongoing mental health monitoring and support systems tailored to the needs of high-risk groups within healthcare environments.

## Conclusion

Despite certain limitations, this study provides important insights into the lasting psychological effects of the COVID-19 pandemic on students and technical staff in healthcare institutions. These findings indicate that younger individuals and females continue to face significant mental health challenges—particularly symptoms of anxiety, depression, and stress—well beyond the acute phase of the pandemic.

These results underscore the urgent need for sustainable mental health support systems. Governments, academic institutions, and healthcare organizations must work collaboratively to develop and implement accessible, evidence-based psychological care strategies. Universities can play a key role by offering ongoing mental health services and digital education programs that extend support to students in remote or underserved areas. Partnerships with internet service providers, along with financial aid in the form of scholarships or student loans, can help ensure equitable access to these resources.

In addition, there is a need for community and familial engagement in promoting mental well-being. Encouraging supportive, low-pressure environments at home can help young individuals cope with ongoing stressors related to academic and professional development.

Overall, the findings from this study highlight the importance of a coordinated and long-term approach to addressing the psychological aftermath of the COVID-19 pandemic. By proactively supporting mental health among students and healthcare workers, we can foster resilience and ensure a more prepared, emotionally healthy workforce for the future.

## Ethical approval

Ethical clearance for the study was obtained from Kasturba Medical College and Kasturba Hospital Institutional Ethics committee (IEC:231/2021).

## Informed consent

Consent to participate in the study was obtained and recorded.

## Registration

ECR/146/Inst/KA/2013/RR-19.

## Preregistered data analysis

This study was not preregistered with an independent registry, and no prior data analysis plan was submitted.

## Data Availability

Underlying data are available from Figshare at: https://doi.org/10.6084/m9.figshare.29957747.v1. This project contains the following underlying data:
•
Ethical_Clearance_and_NOC.pdf (Institutional ethical clearance documentation and no-objection certificate from the Principal Investigator) • Questionnaire.pdf (The study’s survey instrument on depression, anxiety, stress, and COVID-19 impact) • Questionnaire_Responses.csv (Raw responses collected from participants) •
Statistical_Analysis_Output.pdf (Detailed outputs from statistical analyses conducted) • Study_Results.pdf (Summary of the analyzed results as presented in the Results section) • SRQR_Checklist.pdf (Standards for Reporting Qualitative Research checklist completed for the study) Data are available under the terms of the Creative Commons Attribution 4.0 International license (CC-BY 4.0).
