# Supplementary material for: Long-term Psychological Impact of the COVID-19 Pandemic: A Cross-Sectional Survey among Students and Technical Staff in Health Care Institutions
Source: F1000Res. 2026 Jan 14;14:862. Originally published 2025 Sep 3. [Version 2] doi: 10.12688/f1000research.169634.2 (PMC12831951; doi:10.12688/f1000research.169634.2)
Supplement: Peer review history

# Reviewer report: Reviewer response for version 2

**DOI:** 10.5256/f1000research.195093.r452998

**Published:** 2026-02-13

**Authors:** Claudia Chiarolanza

**Affiliations:** 1 Department of Dynamic and Clinical Psychology and Health Studies, Sapienza - University of Rome, Rome, Italy

Thanks for the opportunity to read this article.

 First of all, the introduction doesn't clearly distinguish the impact of COVID-19 on the general population, which could differ for students and trainees, at least according to the Authors. So, this goal should be clearly stated in the introduction and the conclusion. I would suggest extending the theoretical background to the studies elaborated by Arnett on emerging adulthood.

 Regarding the statistical analyses, the chi-square value is related to the number of groups; therefore, I suggest checking the adjusted residuals to verify whether the significant difference is solely due to sample size. There is a lack of clarity about the choice of the sample; so I think that it is important to deepen this aspect.

Is the work clearly and accurately presented and does it cite the current literature?

Partly

If applicable, is the statistical analysis and its interpretation appropriate?

Partly

Are all the source data underlying the results available to ensure full reproducibility?

Partly

Is the study design appropriate and is the work technically sound?

Partly

Are the conclusions drawn adequately supported by the results?

Partly

Are sufficient details of methods and analysis provided to allow replication by others?

Partly

Reviewer Expertise:

Quantitative and qualitative methodology, close relationships, emerging adulthood, coping strategies

I confirm that I have read this submission and believe that I have an appropriate level of expertise to state that I do not consider it to be of an acceptable scientific standard, for reasons outlined above.

# Reviewer report: Reviewer response for version 2

**DOI:** 10.5256/f1000research.195093.r413192

**Published:** 2026-01-24

**Authors:** Rachana Prabhu

**Affiliations:** 1 Yenepoya Dental College, Mangaluru, Karnataka, India

This research article explores the long-term psychological impact of the COVID-19 pandemic on a defined population using a questionnaire-based cross-sectional design. The study addresses a timely and clinically relevant issue, particularly in the context of prolonged pandemic-related stressors. The authors present descriptive and inferential analyses to identify levels of anxiety, depression, and stress, and attempt to correlate these outcomes with demographic variables. The findings provide useful insight into the persistent mental health burden associated with COVID-19 and underscore the need for targeted psychological support strategies. The manuscript is generally well organized, and the results are clearly presented, contributing to the growing body of post-pandemic mental health literature

**Positives:**

- **Relevant and timely topic:** The long-term psychological effects of COVID-19 remain an important area of investigation.
- **Clear objective:** The aim of evaluating psychological impact using standardized measures is well defined.
- **Use of validated assessment tools:** Established scales enhance the credibility of the findings.
- **Structured presentation:** The manuscript follows a logical flow from introduction to conclusion.

**Negatives:**

- **Cross-sectional design:** Limits causal inference regarding psychological outcomes.
- **Limited discussion of confounding factors:** Socioeconomic status and pre-existing mental health conditions could have been explored further.

Is the work clearly and accurately presented and does it cite the current literature?

Yes

If applicable, is the statistical analysis and its interpretation appropriate?

Yes

Are all the source data underlying the results available to ensure full reproducibility?

Yes

Is the study design appropriate and is the work technically sound?

Yes

Are the conclusions drawn adequately supported by the results?

Yes

Are sufficient details of methods and analysis provided to allow replication by others?

Yes

Reviewer Expertise:

Public health, healthcare research, clinical sciences

I confirm that I have read this submission and believe that I have an appropriate level of expertise to confirm that it is of an acceptable scientific standard.

# Reviewer report: Reviewer response for version 1

**DOI:** 10.5256/f1000research.186992.r413194

**Published:** 2025-10-15

**Authors:** Aman Chowdhry

**Affiliations:** 1 Sharda University, Greater Noida, Uttar Pradesh, India

The manuscript, although submitted somewhat later than ideal, contributes valuable insights into the sustained psychological sequelae of the COVID-19 pandemic among healthcare-associated academic and technical populations. The study is relevant and timely in exploring long-term psychological outcomes, particularly among groups often underrepresented in post-pandemic mental health research.

 The methodology is generally well-described, but would benefit from further clarity and structuring according to the PICO framework, as follows:

 P (Population): Clearly define the population — specify which categories of students (undergraduate, postgraduate, interns) and technical staff (laboratory technicians, administrative support, nursing aides, etc.) were included. Additionally, identify the nature of the healthcare institutions (medical, dental, nursing, or allied health) to enhance context and reproducibility.

 I (Intervention): The use of DASS-21 and IES-R scales is appropriate for assessing depression, anxiety, stress, and trauma-related distress. However, the authors should include a short rationale for employing both instruments concurrently, explaining how they complement each other in evaluating different but overlapping aspects of psychological impact.

 C (Comparator): The absence of baseline (pre-pandemic) data is a key limitation. Without a comparator, attributing observed mental health outcomes solely to COVID-19 is difficult. The manuscript should explicitly acknowledge this gap and discuss how potential confounding variables (e.g., prior mental health history, socioeconomic status, or workload differences) were addressed or at least recognised.

 O (Outcome): The sample size (n = 250), while adequate for a preliminary cross-sectional assessment, limits the generalizability of findings. If participants were drawn from a single or limited number of institutions, this should be stated as a limitation. Furthermore, as a cross-sectional design, the study captures only a single time-point, preventing conclusions about causality or long-term trends.

 Lastly, although the manuscript addresses an important topic, **COVID-19 has been extensively studied in recent years**. To strengthen the relevance and currency of this work, it is recommended to incorporate at least two recent publications (published between 2023 and 2025) that align with the study’s focus on the *long-term psychological impact* of the pandemic.

 Overall Recommendation: The study holds merit for indexing after methodological refinement and clarification of the PICO elements. The limitations — particularly the lack of baseline data, potential sampling bias, and absence of longitudinal follow-up — should be clearly acknowledged in the Discussion section to strengthen the manuscript’s transparency and scientific rigour.

Is the work clearly and accurately presented and does it cite the current literature?

Partly

If applicable, is the statistical analysis and its interpretation appropriate?

Partly

Are all the source data underlying the results available to ensure full reproducibility?

Partly

Is the study design appropriate and is the work technically sound?

Yes

Are the conclusions drawn adequately supported by the results?

Yes

Are sufficient details of methods and analysis provided to allow replication by others?

Yes

Reviewer Expertise:

I have previously published papers on COVID-19 and healthcare, including work on “Strengthening Health Care Research and Academics During and After the COVID-19 Pandemic.” In one of my articles, I have also discussed the evolving challenges and opportunities faced by healthcare institutions in the post-pandemic era.

I confirm that I have read this submission and believe that I have an appropriate level of expertise to confirm that it is of an acceptable scientific standard, however I have significant reservations, as outlined above.

# Response

**Published:** 2026-01-09

**Authors:** Ann Sales

**Affiliations:** Prosthodontics, Manipal Academy of Higher Education, Manipal, Karnataka, India

We thank the reviewer for their careful evaluation of our manuscript and for the constructive suggestions that have helped strengthen the clarity, transparency, and methodological rigour of the study. We have addressed each point in detail below and revised the manuscript accordingly.

**1. Clarification of Population (PICO – Population)**

**Reviewer comment:**

 The population should be clearly defined, including student categories, technical staff roles, and the nature of healthcare institutions.

**Response:**

 We have revised the *Materials & Methods* section to explicitly define the study population. Student participants are now clearly described as undergraduate students, postgraduate students, and interns. Technical staff are specified as laboratory technicians, clinical assistants, and administrative support personnel involved in healthcare education or delivery. Additionally, the participating institutions are described as healthcare-related academic institutions, and the limited institutional setting has been acknowledged as a factor affecting generalizability.

**2. Rationale for Use of DASS-21 and IES-R (Intervention)**

**Reviewer comment:**

 A rationale should be provided for the concurrent use of DASS-21 and IES-R.

**Response:**

 We have added a clear justification in the *Materials & Methods* section explaining that DASS-21 was used to assess generalized emotional states (depression, anxiety, and stress), while the IES-R was employed to capture trauma-related distress linked specifically to the COVID-19 pandemic. The complementary nature of these instruments is now explicitly stated, highlighting their combined value in providing a comprehensive assessment of long-term psychological impact.

**3. Absence of Baseline Data and Confounders (Comparator)**

**Reviewer comment:**

 The lack of pre-pandemic baseline data should be acknowledged, and potential confounders should be discussed.

**Response:**

 We have explicitly acknowledged the absence of baseline pre-pandemic mental health data in both the *Materials & Methods* and *Limitations* sections. The manuscript now clarifies that findings should be interpreted as indicative of persistent psychological symptoms rather than causal effects. We have also discussed unmeasured confounding factors, including prior mental health status, socioeconomic variables, and workload differences, and acknowledged their potential influence on outcomes.

**4. Sample Size, Generalizability, and Study Design (Outcome)**

**Reviewer comment:**

 Limitations related to sample size, institutional setting, and cross-sectional design should be stated.

**Response:**

 The manuscript has been revised to clarify that the sample size (n = 250) is adequate for a cross-sectional analysis but limits broader generalizability. The cross-sectional design and its inability to assess causality or longitudinal trends are now clearly discussed in the *Limitations* section.

**Overall Response**

 We believe that these revisions have improved the clarity, methodological transparency, and interpretative balance of the manuscript. The limitations are now explicitly acknowledged, the rationale for methodological choices is clearer, and the study’s contribution to understanding long-term psychological effects of COVID-19 among healthcare-associated populations is more clearly articulated.

# Reviewer report: Reviewer response for version 1

**DOI:** 10.5256/f1000research.186992.r413188

**Published:** 2025-09-24

**Authors:** Shruti Sunil

**Affiliations:** 1 Qatar University, Doha, Qatar

The manuscript is well written, comprehensive, and makes a valuable contribution to the field that addresses an important issue. Its impact could be further enhanced by discussing potential directions for future research, particularly in relation to the acknowledged limitations. In addition, refining the conclusion to emphasize concrete, practical recommendations would strengthen the study’s applicability and provide clearer guidance.

Is the work clearly and accurately presented and does it cite the current literature?

Yes

If applicable, is the statistical analysis and its interpretation appropriate?

Yes

Are all the source data underlying the results available to ensure full reproducibility?

Yes

Is the study design appropriate and is the work technically sound?

Yes

Are the conclusions drawn adequately supported by the results?

Yes

Are sufficient details of methods and analysis provided to allow replication by others?

Yes

Reviewer Expertise:

Medical education, Epidemiology, QoL, AI in medical and dental research

I confirm that I have read this submission and believe that I have an appropriate level of expertise to confirm that it is of an acceptable scientific standard.
